# Mixed higher-order anisotropic flow and nonlinear response coefficients of charged particles in $\mathrm {PbPb}$ collisions at $\sqrt{\smash [b]{s_{_{\mathrm {NN}}}}} = 2.76$ and 5.02$\,\text {TeV}$

**DOI:** 10.1140/epjc/s10052-020-7834-9

**Published:** 2020-06-13

**Authors:** A. M. Sirunyan, A. Tumasyan, W. Adam, F. Ambrogi, T. Bergauer, J. Brandstetter, M. Dragicevic, J. Erö, A. Escalante Del Valle, M. Flechl, R. Frühwirth, M. Jeitler, N. Krammer, I. Krätschmer, D. Liko, T. Madlener, I. Mikulec, N. Rad, J. Schieck, R. Schöfbeck, M. Spanring, D. Spitzbart, W. Waltenberger, C.-E. Wulz, M. Zarucki, V. Drugakov, V. Mossolov, J. Suarez Gonzalez, M. R. Darwish, E. A. De Wolf, D. Di Croce, X. Janssen, A. Lelek, M. Pieters, H. Rejeb Sfar, H. Van Haevermaet, P. Van Mechelen, S. Van Putte, N. Van Remortel, F. Blekman, E. S. Bols, S. S. Chhibra, J. D’Hondt, J. De Clercq, D. Lontkovskyi, S. Lowette, I. Marchesini, S. Moortgat, L. Moreels, Q. Python, K. Skovpen, S. Tavernier, W. Van Doninck, P. Van Mulders, I. Van Parijs, D. Beghin, B. Bilin, H. Brun, B. Clerbaux, G. De Lentdecker, H. Delannoy, B. Dorney, L. Favart, A. Grebenyuk, A. K. Kalsi, A. Popov, N. Postiau, E. Starling, L. Thomas, C. Vander Velde, P. Vanlaer, D. Vannerom, T. Cornelis, D. Dobur, I. Khvastunov, M. Niedziela, C. Roskas, D. Trocino, M. Tytgat, W. Verbeke, B. Vermassen, M. Vit, N. Zaganidis, O. Bondu, G. Bruno, C. Caputo, P. David, C. Delaere, M. Delcourt, A. Giammanco, V. Lemaitre, A. Magitteri, J. Prisciandaro, A. Saggio, M. Vidal Marono, P. Vischia, J. Zobec, F. L. Alves, G. A. Alves, G. Correia Silva, C. Hensel, A. Moraes, P. Rebello Teles, E. Belchior Batista Das Chagas, W. Carvalho, J. Chinellato, E. Coelho, E. M. Da Costa, G. G. Da Silveira, D. De Jesus Damiao, C. De Oliveira Martins, S. Fonseca De Souza, L. M. Huertas Guativa, H. Malbouisson, J. Martins, D. Matos Figueiredo, M. Medina Jaime, M. Melo De Almeida, C. Mora Herrera, L. Mundim, H. Nogima, W. L. Prado Da Silva, L. J. Sanchez Rosas, A. Santoro, A. Sznajder, M. Thiel, E. J. Tonelli Manganote, F. Torres Da Silva De Araujo, A. Vilela Pereira, C. A. Bernardes, L. Calligaris, T. R. Fernandez Perez Tomei, E. M. Gregores, D. S. Lemos, P. G. Mercadante, S. F. Novaes, S. S. Padula, A. Aleksandrov, G. Antchev, R. Hadjiiska, P. Iaydjiev, A. Marinov, M. Misheva, M. Rodozov, M. Shopova, G. Sultanov, M. Bonchev, A. Dimitrov, T. Ivanov, L. Litov, B. Pavlov, P. Petkov, W. Fang, X. Gao, L. Yuan, M. Ahmad, G. M. Chen, H. S. Chen, M. Chen, C. H. Jiang, D. Leggat, H. Liao, Z. Liu, S. M. Shaheen, A. Spiezia, J. Tao, E. Yazgan, H. Zhang, S. Zhang, J. Zhao, A. Agapitos, Y. Ban, G. Chen, A. Levin, J. Li, L. Li, Q. Li, Y. Mao, S. J. Qian, D. Wang, Q. Wang, Z. Hu, Y. Wang, C. Avila, A. Cabrera, L. F. Chaparro Sierra, C. Florez, C. F. González Hernández, M. A. Segura Delgado, J. Mejia Guisao, J. D. Ruiz Alvarez, C. A. Salazar González, N. Vanegas Arbelaez, D. Giljanović, N. Godinovic, D. Lelas, I. Puljak, T. Sculac, Z. Antunovic, M. Kovac, V. Brigljevic, S. Ceci, D. Ferencek, K. Kadija, B. Mesic, M. Roguljic, A. Starodumov, T. Susa, M. W. Ather, A. Attikis, E. Erodotou, A. Ioannou, M. Kolosova, S. Konstantinou, G. Mavromanolakis, J. Mousa, C. Nicolaou, F. Ptochos, P. A. Razis, H. Rykaczewski, D. Tsiakkouri, M. Finger, M. Finger, A. Kveton, J. Tomsa, E. Ayala, E. Carrera Jarrin, Y. Assran, S. Elgammal, S. Bhowmik, A. Carvalho Antunes De Oliveira, R. K. Dewanjee, K. Ehataht, M. Kadastik, M. Raidal, C. Veelken, P. Eerola, L. Forthomme, H. Kirschenmann, K. Osterberg, M. Voutilainen, F. Garcia, J. Havukainen, J. K. Heikkilä, T. Järvinen, V. Karimäki, M. S. Kim, R. Kinnunen, T. Lampén, K. Lassila-Perini, S. Laurila, S. Lehti, T. Lindén, P. Luukka, T. Mäenpää, H. Siikonen, E. Tuominen, J. Tuominiemi, T. Tuuva, M. Besancon, F. Couderc, M. Dejardin, D. Denegri, B. Fabbro, J. L. Faure, F. Ferri, S. Ganjour, A. Givernaud, P. Gras, G. Hamel de Monchenault, P. Jarry, C. Leloup, E. Locci, J. Malcles, J. Rander, A. Rosowsky, M. Ö. Sahin, A. Savoy-Navarro, M. Titov, S. Ahuja, C. Amendola, F. Beaudette, P. Busson, C. Charlot, B. Diab, G. Falmagne, R. Granier de Cassagnac, I. Kucher, A. Lobanov, C. Martin Perez, M. Nguyen, C. Ochando, P. Paganini, J. Rembser, R. Salerno, J. B. Sauvan, Y. Sirois, A. Zabi, A. Zghiche, J.-L. Agram, J. Andrea, D. Bloch, G. Bourgatte, J.-M. Brom, E. C. Chabert, C. Collard, E. Conte, J.-C. Fontaine, D. Gelé, U. Goerlach, M. Jansová, A.-C. Le Bihan, N. Tonon, P. Van Hove, S. Gadrat, S. Beauceron, C. Bernet, G. Boudoul, C. Camen, N. Chanon, R. Chierici, D. Contardo, P. Depasse, H. El Mamouni, J. Fay, S. Gascon, M. Gouzevitch, B. Ille, S. Jain, F. Lagarde, I. B. Laktineh, H. Lattaud, A. Lesauvage, M. Lethuillier, L. Mirabito, S. Perries, V. Sordini, L. Torterotot, G. Touquet, M. Vander Donckt, S. Viret, A. Khvedelidze, Z. Tsamalaidze, C. Autermann, L. Feld, M. K. Kiesel, K. Klein, M. Lipinski, D. Meuser, A. Pauls, M. Preuten, M. P. Rauch, C. Schomakers, J. Schulz, M. Teroerde, B. Wittmer, A. Albert, M. Erdmann, S. Erdweg, T. Esch, B. Fischer, R. Fischer, S. Ghosh, T. Hebbeker, K. Hoepfner, H. Keller, L. Mastrolorenzo, M. Merschmeyer, A. Meyer, P. Millet, G. Mocellin, S. Mondal, S. Mukherjee, D. Noll, A. Novak, T. Pook, A. Pozdnyakov, T. Quast, M. Radziej, Y. Rath, H. Reithler, M. Rieger, J. Roemer, A. Schmidt, S. C. Schuler, A. Sharma, S. Thüer, S. Wiedenbeck, S. Zaleski, G. Flügge, W. Haj Ahmad, O. Hlushchenko, T. Kress, T. Müller, A. Nehrkorn, A. Nowack, C. Pistone, O. Pooth, D. Roy, H. Sert, A. Stahl, M. Aldaya Martin, P. Asmuss, I. Babounikau, H. Bakhshiansohi, K. Beernaert, O. Behnke, U. Behrens, A. Bermúdez Martínez, D. Bertsche, A. A. Bin Anuar, K. Borras, V. Botta, A. Campbell, A. Cardini, P. Connor, S. Consuegra Rodríguez, C. Contreras-Campana, V. Danilov, A. De Wit, M. M. Defranchis, C. Diez Pardos, D. Domínguez Damiani, G. Eckerlin, D. Eckstein, T. Eichhorn, A. Elwood, E. Eren, E. Gallo, A. Geiser, J. M. Grados Luyando, A. Grohsjean, M. Guthoff, M. Haranko, A. Harb, A. Jafari, N. Z. Jomhari, H. Jung, A. Kasem, M. Kasemann, H. Kaveh, J. Keaveney, C. Kleinwort, J. Knolle, D. Krücker, W. Lange, T. Lenz, J. Leonard, J. Lidrych, K. Lipka, W. Lohmann, R. Mankel, I.-A. Melzer-Pellmann, A. B. Meyer, M. Meyer, M. Missiroli, G. Mittag, J. Mnich, A. Mussgiller, V. Myronenko, D. Pérez Adán, S. K. Pflitsch, D. Pitzl, A. Raspereza, A. Saibel, M. Savitskyi, V. Scheurer, P. Schütze, C. Schwanenberger, R. Shevchenko, A. Singh, H. Tholen, O. Turkot, A. Vagnerini, M. Van De Klundert, G. P. Van Onsem, R. Walsh, Y. Wen, K. Wichmann, C. Wissing, O. Zenaiev, R. Zlebcik, R. Aggleton, S. Bein, L. Benato, A. Benecke, V. Blobel, T. Dreyer, A. Ebrahimi, A. Fröhlich, C. Garbers, E. Garutti, D. Gonzalez, P. Gunnellini, J. Haller, A. Hinzmann, A. Karavdina, G. Kasieczka, R. Klanner, R. Kogler, N. Kovalchuk, S. Kurz, V. Kutzner, J. Lange, T. Lange, A. Malara, J. Multhaup, C. E. N. Niemeyer, A. Perieanu, A. Reimers, O. Rieger, C. Scharf, P. Schleper, S. Schumann, J. Schwandt, J. Sonneveld, H. Stadie, G. Steinbrück, F. M. Stober, M. Stöver, B. Vormwald, I. Zoi, M. Akbiyik, C. Barth, M. Baselga, S. Baur, T. Berger, E. Butz, R. Caspart, T. Chwalek, W. De Boer, A. Dierlamm, K. El Morabit, N. Faltermann, M. Giffels, P. Goldenzweig, A. Gottmann, M. A. Harrendorf, F. Hartmann, U. Husemann, S. Kudella, S. Mitra, M. U. Mozer, D. Müller, Th. Müller, M. Musich, A. Nürnberg, G. Quast, K. Rabbertz, M. Schröder, I. Shvetsov, H. J. Simonis, R. Ulrich, M. Wassmer, M. Weber, C. Wöhrmann, R. Wolf, G. Anagnostou, P. Asenov, G. Daskalakis, T. Geralis, A. Kyriakis, D. Loukas, G. Paspalaki, M. Diamantopoulou, G. Karathanasis, P. Kontaxakis, A. Manousakis-katsikakis, A. Panagiotou, I. Papavergou, N. Saoulidou, A. Stakia, K. Theofilatos, K. Vellidis, E. Vourliotis, G. Bakas, K. Kousouris, I. Papakrivopoulos, G. Tsipolitis, I. Evangelou, C. Foudas, P. Gianneios, P. Katsoulis, P. Kokkas, S. Mallios, K. Manitara, N. Manthos, I. Papadopoulos, J. Strologas, F. A. Triantis, D. Tsitsonis, M. Bartók, R. Chudasama, M. Csanad, P. Major, K. Mandal, A. Mehta, M. I. Nagy, G. Pasztor, O. Surányi, G. I. Veres, G. Bencze, C. Hajdu, D. Horvath, F. Sikler, T. Vámi, V. Veszpremi, G. Vesztergombi, N. Beni, S. Czellar, J. Karancsi, A. Makovec, J. Molnar, Z. Szillasi, P. Raics, D. Teyssier, Z. L. Trocsanyi, B. Ujvari, T. Csorgo, W. J. Metzger, F. Nemes, T. Novak, S. Choudhury, J. R. Komaragiri, P. C. Tiwari, S. Bahinipati, C. Kar, G. Kole, P. Mal, V. K. Muraleedharan Nair Bindhu, A. Nayak, D. K. Sahoo, S. K. Swain, S. Bansal, S. B. Beri, V. Bhatnagar, S. Chauhan, R. Chawla, N. Dhingra, R. Gupta, A. Kaur, M. Kaur, S. Kaur, P. Kumari, M. Lohan, M. Meena, K. Sandeep, S. Sharma, J. B. Singh, A. K. Virdi, G. Walia, A. Bhardwaj, B. C. Choudhary, R. B. Garg, M. Gola, S. Keshri, A. Kumar, S. Malhotra, M. Naimuddin, P. Priyanka, K. Ranjan, A. Shah, R. Sharma, R. Bhardwaj, M. Bharti, R. Bhattacharya, S. Bhattacharya, U. Bhawandeep, D. Bhowmik, S. Dey, S. Dutta, S. Ghosh, M. Maity, K. Mondal, S. Nandan, A. Purohit, P. K. Rout, G. Saha, S. Sarkar, T. Sarkar, M. Sharan, B. Singh, S. Thakur, P. K. Behera, P. Kalbhor, A. Muhammad, P. R. Pujahari, A. Sharma, A. K. Sikdar, D. Dutta, V. Jha, V. Kumar, D. K. Mishra, P. K. Netrakanti, L. M. Pant, P. Shukla, T. Aziz, M. A. Bhat, S. Dugad, G. B. Mohanty, N. Sur, R. K. Verma, S. Banerjee, S. Bhattacharya, S. Chatterjee, P. Das, M. Guchait, S. Karmakar, S. Kumar, G. Majumder, K. Mazumdar, N. Sahoo, S. Sawant, S. Chauhan, S. Dube, V. Hegde, B. Kansal, A. Kapoor, K. Kothekar, S. Pandey, A. Rane, A. Rastogi, S. Sharma, S. Chenarani, E. Eskandari Tadavani, S. M. Etesami, M. Khakzad, M. Mohammadi Najafabadi, M. Naseri, F. Rezaei Hosseinabadi, M. Felcini, M. Grunewald, M. Abbrescia, R. Aly, C. Calabria, A. Colaleo, D. Creanza, L. Cristella, N. De Filippis, M. De Palma, A. Di Florio, L. Fiore, A. Gelmi, G. Iaselli, M. Ince, S. Lezki, G. Maggi, M. Maggi, G. Miniello, S. My, S. Nuzzo, A. Pompili, G. Pugliese, R. Radogna, A. Ranieri, G. Selvaggi, L. Silvestris, R. Venditti, P. Verwilligen, G. Abbiendi, C. Battilana, D. Bonacorsi, L. Borgonovi, S. Braibant-Giacomelli, R. Campanini, P. Capiluppi, A. Castro, F. R. Cavallo, C. Ciocca, G. Codispoti, M. Cuffiani, G. M. Dallavalle, F. Fabbri, A. Fanfani, E. Fontanesi, P. Giacomelli, C. Grandi, L. Guiducci, F. Iemmi, S. Lo Meo, S. Marcellini, G. Masetti, F. L. Navarria, A. Perrotta, F. Primavera, A. M. Rossi, T. Rovelli, G. P. Siroli, N. Tosi, S. Albergo, S. Costa, A. Di Mattia, R. Potenza, A. Tricomi, C. Tuve, G. Barbagli, R. Ceccarelli, K. Chatterjee, V. Ciulli, C. Civinini, R. D’Alessandro, E. Focardi, G. Latino, P. Lenzi, M. Meschini, S. Paoletti, G. Sguazzoni, D. Strom, L. Viliani, L. Benussi, S. Bianco, D. Piccolo, M. Bozzo, F. Ferro, R. Mulargia, E. Robutti, S. Tosi, A. Benaglia, A. Beschi, F. Brivio, V. Ciriolo, S. Di Guida, M. E. Dinardo, P. Dini, S. Gennai, A. Ghezzi, P. Govoni, L. Guzzi, M. Malberti, S. Malvezzi, D. Menasce, F. Monti, L. Moroni, G. Ortona, M. Paganoni, D. Pedrini, S. Ragazzi, T. Tabarelli de Fatis, D. Zuolo, S. Buontempo, N. Cavallo, A. De Iorio, A. Di Crescenzo, F. Fabozzi, F. Fienga, G. Galati, A. O. M. Iorio, L. Lista, S. Meola, P. Paolucci, B. Rossi, C. Sciacca, E. Voevodina, P. Azzi, N. Bacchetta, D. D.Bisello, A. Boletti, A. Bragagnolo, R. Carlin, P. Checchia, P. De Castro Manzano, T. Dorigo, U. Dosselli, F. Gasparini, U. Gasparini, A. Gozzelino, S. Y. Hoh, P. Lujan, M. Margoni, A. T. Meneguzzo, J. Pazzini, M. Presilla, P. Ronchese, R. Rossin, F. Simonetto, A. Tiko, M. Tosi, M. Zanetti, P. Zotto, G. Zumerle, A. Braghieri, P. Montagna, S. P. Ratti, V. Re, M. Ressegotti, C. Riccardi, P. Salvini, I. Vai, P. Vitulo, M. Biasini, G. M. Bilei, D. Ciangottini, L. Fanò, P. Lariccia, R. Leonardi, G. Mantovani, V. Mariani, M. Menichelli, A. Rossi, A. Santocchia, D. Spiga, K. Androsov, P. Azzurri, G. Bagliesi, V. Bertacchi, L. Bianchini, T. Boccali, R. Castaldi, M. A. Ciocci, R. Dell’Orso, G. Fedi, L. Giannini, A. Giassi, M. T. Grippo, F. Ligabue, E. Manca, G. Mandorli, A. Messineo, F. Palla, A. Rizzi, G. Rolandi, S. Roy Chowdhury, A. Scribano, P. Spagnolo, R. Tenchini, G. Tonelli, N. Turini, A. Venturi, P. G. Verdini, F. Cavallari, M. Cipriani, D. Del Re, E. Di Marco, M. Diemoz, E. Longo, B. Marzocchi, P. Meridiani, G. Organtini, F. Pandolfi, R. Paramatti, C. Quaranta, S. Rahatlou, C. Rovelli, F. Santanastasio, L. Soffi, N. Amapane, R. Arcidiacono, S. Argiro, M. Arneodo, N. Bartosik, R. Bellan, C. Biino, A. Cappati, N. Cartiglia, S. Cometti, M. Costa, R. Covarelli, N. Demaria, B. Kiani, C. Mariotti, S. Maselli, E. Migliore, V. Monaco, E. Monteil, M. Monteno, M. M. Obertino, L. Pacher, N. Pastrone, M. Pelliccioni, G. L. Pinna Angioni, A. Romero, M. Ruspa, R. Sacchi, R. Salvatico, V. Sola, A. Solano, D. Soldi, A. Staiano, S. Belforte, V. Candelise, M. Casarsa, F. Cossutti, A. Da Rold, G. Della Ricca, F. Vazzoler, A. Zanetti, B. Kim, D. H. Kim, G. N. Kim, J. Lee, S. W. Lee, C. S. Moon, Y. D. Oh, S. I. Pak, S. Sekmen, D. C. Son, Y. C. Yang, H. Kim, D. H. Moon, G. Oh, B. Francois, T. J. Kim, J. Park, S. Cho, S. Choi, Y. Go, D. Gyun, S. Ha, B. Hong, K. Lee, K. S. Lee, J. Lim, J. Park, S. K. Park, Y. Roh, J. Yoo, J. Goh, H. S. Kim, J. Almond, J. H. Bhyun, J. Choi, S. Jeon, J. Kim, J. S. Kim, H. Lee, K. Lee, S. Lee, K. Nam, M. Oh, S. B. Oh, B. C. Radburn-Smith, U. K. Yang, H. D. Yoo, I. Yoon, G. B. Yu, D. Jeon, H. Kim, J. H. Kim, J. S. H. Lee, I. C. Park, I. Watson, Y. Choi, C. Hwang, Y. Jeong, J. Lee, Y. Lee, I. Yu, V. Veckalns, V. Dudenas, A. Juodagalvis, G. Tamulaitis, J. Vaitkus, Z. A. Ibrahim, F. Mohamad Idris, W. A. T. Wan Abdullah, M. N. Yusli, Z. Zolkapli, J. F. Benitez, A. Castaneda Hernandez, J. A. Murillo Quijada, L. Valencia Palomo, H. Castilla-Valdez, E. De La Cruz-Burelo, I. Heredia-De La Cruz, R. Lopez-Fernandez, A. Sanchez-Hernandez, S. Carrillo Moreno, C. Oropeza Barrera, M. Ramirez-Garcia, F. Vazquez Valencia, J. Eysermans, I. Pedraza, H. A. Salazar Ibarguen, C. Uribe Estrada, A. Morelos Pineda, N. Raicevic, D. Krofcheck, S. Bheesette, P. H. Butler, A. Ahmad, M. Ahmad, Q. Hassan, H. R. Hoorani, W. A. Khan, M. A. Shah, M. Shoaib, M. Waqas, V. Avati, L. Grzanka, M. Malawski, H. Bialkowska, M. Bluj, B. Boimska, M. Górski, M. Kazana, M. Szleper, P. Zalewski, K. Bunkowski, A. Byszuk, K. Doroba, A. Kalinowski, M. Konecki, J. Krolikowski, M. Misiura, M. Olszewski, A. Pyskir, M. Walczak, M. Araujo, P. Bargassa, D. Bastos, A. Di Francesco, P. Faccioli, B. Galinhas, M. Gallinaro, J. Hollar, N. Leonardo, J. Seixas, K. Shchelina, G. Strong, O. Toldaiev, J. Varela, S. Afanasiev, P. Bunin, M. Gavrilenko, I. Golutvin, I. Gorbunov, A. Kamenev, V. Karjavine, A. Lanev, A. Malakhov, V. Matveev, P. Moisenz, V. Palichik, V. Perelygin, M. Savina, S. Shmatov, S. Shulha, N. Skatchkov, V. Smirnov, N. Voytishin, A. Zarubin, L. Chtchipounov, V. Golovtcov, Y. Ivanov, V. Kim, E. Kuznetsova, P. Levchenko, V. Murzin, V. Oreshkin, I. Smirnov, D. Sosnov, V. Sulimov, L. Uvarov, A. Vorobyev, Yu. Andreev, A. Dermenev, S. Gninenko, N. Golubev, A. Karneyeu, M. Kirsanov, N. Krasnikov, A. Pashenkov, D. Tlisov, A. Toropin, V. Epshteyn, V. Gavrilov, N. Lychkovskaya, A. Nikitenko, V. Popov, I. Pozdnyakov, G. Safronov, A. Spiridonov, A. Stepennov, M. Toms, E. Vlasov, A. Zhokin, T. Aushev, O. Bychkova, R. Chistov, M. Danilov, S. Polikarpov, E. Tarkovskii, V. Andreev, M. Azarkin, I. Dremin, M. Kirakosyan, A. Terkulov, A. Belyaev, E. Boos, A. Ershov, A. Gribushin, A. Kaminskiy, O. Kodolova, V. Korotkikh, I. Lokhtin, S. Obraztsov, S. Petrushanko, V. Savrin, A. Snigirev, I. Vardanyan, A. Barnyakov, V. Blinov, T. Dimova, L. Kardapoltsev, Y. Skovpen, I. Azhgirey, I. Bayshev, S. Bitioukov, V. Kachanov, D. Konstantinov, P. Mandrik, V. Petrov, R. Ryutin, S. Slabospitskii, A. Sobol, S. Troshin, N. Tyurin, A. Uzunian, A. Volkov, A. Babaev, A. Iuzhakov, V. Okhotnikov, V. Borchsh, V. Ivanchenko, E. Tcherniaev, P. Adzic, P. Cirkovic, D. Devetak, M. Dordevic, P. Milenovic, J. Milosevic, M. Stojanovic, M. Aguilar-Benitez, J. Alcaraz Maestre, A. lvarez Fernández, I. Bachiller, M. Barrio Luna, J. A. Brochero Cifuentes, C. A. Carrillo Montoya, M. Cepeda, M. Cerrada, N. Colino, B. De La Cruz, A. Delgado Peris, C. Fernandez Bedoya, J. P. Fernández Ramos, J. Flix, M. C. Fouz, O. Gonzalez Lopez, S. Goy Lopez, J. M. Hernandez, M. I. Josa, D. Moran, A. Navarro Tobar, A. Pérez-Calero Yzquierdo, J. Puerta Pelayo, I. Redondo, L. Romero, S. Sánchez Navas, M. S. Soares, A. Triossi, C. Willmott, C. Albajar, J. F. de Trocóniz, B. Alvarez Gonzalez, J. Cuevas, C. Erice, J. Fernandez Menendez, S. Folgueras, I. Gonzalez Caballero, J. R. González Fernández, E. Palencia Cortezon, V. Rodríguez Bouza, S. Sanchez Cruz, I. J. Cabrillo, A. Calderon, B. Chazin Quero, J. Duarte Campderros, M. Fernandez, P. J. Fernández Manteca, A. García Alonso, G. Gomez, C. Martinez Rivero, P. Martinez Ruiz del Arbol, F. Matorras, J. Piedra Gomez, C. Prieels, T. Rodrigo, A. Ruiz-Jimeno, L. Russo, L. Scodellaro, N. Trevisani, I. Vila, J. M. Vizan Garcia, K. Malagalage, W. G. D. Dharmaratna, N. Wickramage, D. Abbaneo, B. Akgun, E. Auffray, G. Auzinger, J. Baechler, P. Baillon, A. H. Ball, D. Barney, J. Bendavid, M. Bianco, A. Bocci, P. Bortignon, E. Bossini, C. Botta, E. Brondolin, T. Camporesi, A. Caratelli, G. Cerminara, E. Chapon, G. Cucciati, D. d’Enterria, A. Dabrowski, N. Daci, V. Daponte, A. David, O. Davignon, A. De Roeck, N. Deelen, M. Deile, M. Dobson, M. Dünser, N. Dupont, A. Elliott-Peisert, F. Fallavollita, D. Fasanella, S. Fiorendi, G. Franzoni, J. Fulcher, W. Funk, S. Giani, D. Gigi, A. Gilbert, K. Gill, F. Glege, M. Gruchala, M. Guilbaud, D. Gulhan, J. Hegeman, C. Heidegger, Y. Iiyama, V. Innocente, P. Janot, O. Karacheban, J. Kaspar, J. Kieseler, M. Krammer, C. Lange, P. Lecoq, C. Lourenço, L. Malgeri, M. Mannelli, A. Massironi, F. Meijers, J. A. Merlin, S. Mersi, E. Meschi, F. Moortgat, M. Mulders, J. Ngadiuba, S. Nourbakhsh, S. Orfanelli, L. Orsini, F. Pantaleo, L. Pape, E. Perez, M. Peruzzi, A. Petrilli, G. Petrucciani, A. Pfeiffer, M. Pierini, F. M. Pitters, D. Rabady, A. Racz, M. Rovere, H. Sakulin, C. Schäfer, C. Schwick, M. Selvaggi, A. Sharma, P. Silva, W. Snoeys, P. Sphicas, J. Steggemann, S. Summers, V. R. Tavolaro, D. Treille, A. Tsirou, A. Vartak, M. Verzetti, W. D. Zeuner, L. Caminada, K. Deiters, W. Erdmann, R. Horisberger, Q. Ingram, H. C. Kaestli, D. Kotlinski, U. Langenegger, T. Rohe, S. A. Wiederkehr, M. Backhaus, P. Berger, N. Chernyavskaya, G. Dissertori, M. Dittmar, M. Donegà, C. Dorfer, T. A. Gómez Espinosa, C. Grab, D. Hits, T. Klijnsma, W. Lustermann, R. A. Manzoni, M. Marionneau, M. T. Meinhard, F. Micheli, P. Musella, F. Nessi-Tedaldi, F. Pauss, G. Perrin, L. Perrozzi, S. Pigazzini, M. G. Ratti, M. Reichmann, C. Reissel, T. Reitenspiess, D. Ruini, D. A. Sanz Becerra, M. Schönenberger, L. Shchutska, M. L. Vesterbacka Olsson, R. Wallny, D. H. Zhu, T. K. Aarrestad, C. Amsler, D. Brzhechko, M. F. Canelli, A. De Cosa, R. Del Burgo, S. Donato, B. Kilminster, S. Leontsinis, V. M. Mikuni, I. Neutelings, G. Rauco, P. Robmann, D. Salerno, K. Schweiger, C. Seitz, Y. Takahashi, S. Wertz, A. Zucchetta, T. H. Doan, C. M. Kuo, W. Lin, A. Roy, S. S. Yu, P. Chang, Y. Chao, K. F. Chen, P. H. Chen, W.-S. Hou, Y. y. Li, R.-S. Lu, E. Paganis, A. Psallidas, A. Steen, B. Asavapibhop, C. Asawatangtrakuldee, N. Srimanobhas, N. Suwonjandee, A. Bat, F. Boran, A. Celik, S. Cerci, S. Damarseckin, Z. S. Demiroglu, F. Dolek, C. Dozen, I. Dumanoglu, G. Gokbulut, E. G. Guler, Y. Guler, I. Hos, C. Isik, E. E. Kangal, O. Kara, A. K. Topaksu, U. Kiminsu, M. Oglakci, G. Onengut, K. Ozdemir, S. Ozturk, A. E. Simsek, D. Sunar Cerci, U. G. Tok, S. Turkcapar, I. S. Zorbakir, C. Zorbilmez, B. Isildak, G. Karapinar, M. Yalvac, I. O. Atakisi, E. Gülmez, M. Kaya, O. Kaya, B. Kaynak, Ö. Özçelik, S. Tekten, E. A. Yetkin, A. Cakir, K. Cankocak, Y. Komurcu, S. Sen, S. Ozkorucuklu, B. Grynyov, L. Levchuk, F. Ball, E. Bhal, S. Bologna, J. J. Brooke, D. Burns, E. Clement, D. Cussans, H. Flacher, J. Goldstein, G. P. Heath, H. F. Heath, L. Kreczko, S. Paramesvaran, B. Penning, T. Sakuma, S. Seif El Nasr-Storey, D. Smith, V. J. Smith, J. Taylor, A. Titterton, K. W. Bell, A. Belyaev, C. Brew, R. M. Brown, D. Cieri, D. J. A. Cockerill, J. A. Coughlan, K. Harder, S. Harper, J. Linacre, K. Manolopoulos, D. M. Newbold, E. Olaiya, D. Petyt, T. Reis, T. Schuh, C. H. Shepherd-Themistocleous, A. Thea, I. R. Tomalin, T. Williams, W. J. Womersley, R. Bainbridge, P. Bloch, J. Borg, S. Breeze, O. Buchmuller, A. Bundock, G. S. Chahal, D. Colling, P. Dauncey, G. Davies, M. Della Negra, R. Di Maria, P. Everaerts, G. Hall, G. Iles, T. James, M. Komm, C. Laner, L. Lyons, A.-M. Magnan, S. Malik, A. Martelli, V. Milosevic, J. Nash, V. Palladino, M. Pesaresi, D. M. Raymond, A. Richards, A. Rose, E. Scott, C. Seez, A. Shtipliyski, M. Stoye, T. Strebler, A. Tapper, K. Uchida, T. Virdee, N. Wardle, D. Winterbottom, J. Wright, A. G. Zecchinelli, S. C. Zenz, J. E. Cole, P. R. Hobson, A. Khan, P. Kyberd, C. K. Mackay, A. Morton, I. D. Reid, L. Teodorescu, S. Zahid, K. Call, J. Dittmann, K. Hatakeyama, C. Madrid, B. McMaster, N. Pastika, C. Smith, R. Bartek, A. Dominguez, R. Uniyal, A. Buccilli, S. I. Cooper, C. Henderson, P. Rumerio, C. West, D. Arcaro, Z. Demiragli, D. Gastler, S. Girgis, D. Pinna, C. Richardson, J. Rohlf, D. Sperka, I. Suarez, L. Sulak, D. Zou, G. Benelli, B. Burkle, X. Coubez, D. Cutts, Y. t. Duh, M. Hadley, J. Hakala, U. Heintz, J. M. Hogan, K. H. M. Kwok, E. Laird, G. Landsberg, J. Lee, Z. Mao, M. Narain, S. Sagir, R. Syarif, E. Usai, D. Yu, W. Zhang, R. Band, C. Brainerd, R. Breedon, M. Calderon De La Barca Sanchez, M. Chertok, J. Conway, R. Conway, P. T. Cox, R. Erbacher, C. Flores, G. Funk, F. Jensen, W. Ko, O. Kukral, R. Lander, M. Mulhearn, D. Pellett, J. Pilot, M. Shi, D. Taylor, K. Tos, M. Tripathi, Z. Wang, F. Zhang, M. Bachtis, C. Bravo, R. Cousins, A. Dasgupta, A. Florent, J. Hauser, M. Ignatenko, N. Mccoll, W. A. Nash, S. Regnard, D. Saltzberg, C. Schnaible, B. Stone, V. Valuev, K. Burt, Y. Chen, R. Clare, J. W. Gary, S. M. A. Ghiasi Shirazi, G. Hanson, G. Karapostoli, E. Kennedy, O. R. Long, M. Olmedo Negrete, M. I. Paneva, W. Si, L. Wang, H. Wei, S. Wimpenny, B. R. Yates, Y. Zhang, J. G. Branson, P. Chang, S. Cittolin, M. Derdzinski, R. Gerosa, D. Gilbert, B. Hashemi, D. Klein, V. Krutelyov, J. Letts, M. Masciovecchio, S. May, S. Padhi, M. Pieri, V. Sharma, M. Tadel, F. Würthwein, A. Yagil, G. Zevi Della Porta, N. Amin, R. Bhandari, C. Campagnari, M. Citron, V. Dutta, M. Franco Sevilla, L. Gouskos, J. Incandela, B. Marsh, H. Mei, A. Ovcharova, H. Qu, J. Richman, U. Sarica, D. Stuart, S. Wang, D. Anderson, A. Bornheim, O. Cerri, I. Dutta, J. M. Lawhorn, N. Lu, J. Mao, H. B. Newman, T. Q. Nguyen, J. Pata, M. Spiropulu, J. R. Vlimant, S. Xie, Z. Zhang, R. Y. Zhu, M. B. Andrews, T. Ferguson, T. Mudholkar, M. Paulini, M. Sun, I. Vorobiev, M. Weinberg, J. P. Cumalat, W. T. Ford, A. Johnson, E. MacDonald, T. Mulholland, R. Patel, A. Perloff, K. Stenson, K. A. Ulmer, S. R. Wagner, J. Alexander, J. Chaves, Y. Cheng, J. Chu, A. Datta, A. Frankenthal, K. Mcdermott, J. R. Patterson, D. Quach, A. Rinkevicius, A. Ryd, S. M. Tan, Z. Tao, J. Thom, P. Wittich, M. Zientek, S. Abdullin, M. Albrow, M. Alyari, G. Apollinari, A. Apresyan, A. Apyan, S. Banerjee, L. A. T. Bauerdick, A. Beretvas, J. Berryhill, P. C. Bhat, K. Burkett, J. N. Butler, A. Canepa, G. B. Cerati, H. W. K. Cheung, F. Chlebana, M. Cremonesi, J. Duarte, V. D. Elvira, J. Freeman, Z. Gecse, E. Gottschalk, L. Gray, D. Green, S. Grünendahl, O. Gutsche, A. R. Hall, J. Hanlon, R. M. Harris, S. Hasegawa, R. Heller, J. Hirschauer, B. Jayatilaka, S. Jindariani, M. Johnson, U. Joshi, B. Klima, M. J. Kortelainen, B. Kreis, S. Lammel, J. Lewis, D. Lincoln, R. Lipton, M. Liu, T. Liu, J. Lykken, K. Maeshima, J. M. Marraffino, D. Mason, P. McBride, P. Merkel, S. Mrenna, S. Nahn, V. O’Dell, V. Papadimitriou, K. Pedro, C. Pena, G. Rakness, F. Ravera, L. Ristori, B. Schneider, E. Sexton-Kennedy, N. Smith, A. Soha, W. J. Spalding, L. Spiegel, S. Stoynev, J. Strait, N. Strobbe, L. Taylor, S. Tkaczyk, N. V. Tran, L. Uplegger, E. W. Vaandering, C. Vernieri, M. Verzocchi, R. Vidal, M. Wang, H. A. Weber, D. Acosta, P. Avery, D. Bourilkov, A. Brinkerhoff, L. Cadamuro, A. Carnes, V. Cherepanov, D. Curry, F. Errico, R. D. Field, S. V. Gleyzer, B. M. Joshi, M. Kim, J. Konigsberg, A. Korytov, K. H. Lo, P. Ma, K. Matchev, N. Menendez, G. Mitselmakher, D. Rosenzweig, K. Shi, J. Wang, S. Wang, X. Zuo, Y. R. Joshi, T. Adams, A. Askew, S. Hagopian, V. Hagopian, K. F. Johnson, R. Khurana, T. Kolberg, G. Martinez, T. Perry, H. Prosper, C. Schiber, R. Yohay, J. Zhang, M. M. Baarmand, V. Bhopatkar, M. Hohlmann, D. Noonan, M. Rahmani, M. Saunders, F. Yumiceva, M. R. Adams, L. Apanasevich, D. Berry, R. R. Betts, R. Cavanaugh, X. Chen, S. Dittmer, O. Evdokimov, C. E. Gerber, D. A. Hangal, D. J. Hofman, K. Jung, C. Mills, T. Roy, M. B. Tonjes, N. Varelas, J. Viinikainen, H. Wang, X. Wang, Z. Wu, M. Alhusseini, B. Bilki, W. Clarida, K. Dilsiz, S. Durgut, R. P. Gandrajula, M. Haytmyradov, V. Khristenko, O. K. Köseyan, J.-P. Merlo, A. Mestvirishvili, A. Moeller, J. Nachtman, H. Ogul, Y. Onel, F. Ozok, A. Penzo, C. Snyder, E. Tiras, J. Wetzel, B. Blumenfeld, A. Cocoros, N. Eminizer, D. Fehling, L. Feng, A. V. Gritsan, W. T. Hung, P. Maksimovic, J. Roskes, M. Swartz, M. Xiao, C. Baldenegro Barrera, P. Baringer, A. Bean, S. Boren, J. Bowen, A. Bylinkin, T. Isidori, S. Khalil, J. King, G. Krintiras, A. Kropivnitskaya, C. Lindsey, D. Majumder, W. Mcbrayer, N. Minafra, M. Murray, C. Rogan, C. Royon, S. Sanders, E. Schmitz, J. D. Tapia Takaki, Q. Wang, J. Williams, G. Wilson, S. Duric, A. Ivanov, K. Kaadze, D. Kim, Y. Maravin, D. R. Mendis, T. Mitchell, A. Modak, A. Mohammadi, F. Rebassoo, D. Wright, A. Baden, O. Baron, A. Belloni, S. C. Eno, Y. Feng, N. J. Hadley, S. Jabeen, G. Y. Jeng, R. G. Kellogg, J. Kunkle, A. C. Mignerey, S. Nabili, F. Ricci-Tam, M. Seidel, Y. H. Shin, A. Skuja, S. C. Tonwar, K. Wong, D. Abercrombie, B. Allen, A. Baty, R. Bi, S. Brandt, W. Busza, I. A. Cali, M. D’Alfonso, G. Gomez Ceballos, M. Goncharov, P. Harris, D. Hsu, M. Hu, M. Klute, D. Kovalskyi, Y.-J. Lee, P. D. Luckey, B. Maier, A. C. Marini, C. Mcginn, C. Mironov, S. Narayanan, X. Niu, C. Paus, D. Rankin, C. Roland, G. Roland, Z. Shi, G. S. F. Stephans, K. Sumorok, K. Tatar, D. Velicanu, J. Wang, T. W. Wang, B. Wyslouch, A. C. Benvenuti, R. M. Chatterjee, A. Evans, S. Guts, P. Hansen, J. Hiltbrand, Sh. Jain, Y. Kubota, Z. Lesko, J. Mans, R. Rusack, M. A. Wadud, J. G. Acosta, S. Oliveros, K. Bloom, D. R. Claes, C. Fangmeier, L. Finco, F. Golf, R. Gonzalez Suarez, R. Kamalieddin, I. Kravchenko, J. E. Siado, G. R. Snow, B. Stieger, W. Tabb, G. Agarwal, C. Harrington, I. Iashvili, A. Kharchilava, C. McLean, D. Nguyen, A. Parker, J. Pekkanen, S. Rappoccio, B. Roozbahani, G. Alverson, E. Barberis, C. Freer, Y. Haddad, A. Hortiangtham, G. Madigan, D. M. Morse, T. Orimoto, L. Skinnari, A. Tishelman-Charny, T. Wamorkar, B. Wang, A. Wisecarver, D. Wood, S. Bhattacharya, J. Bueghly, T. Gunter, K. A. Hahn, N. Odell, M. H. Schmitt, K. Sung, M. Trovato, M. Velasco, R. Bucci, N. Dev, R. Goldouzian, M. Hildreth, K. Hurtado Anampa, C. Jessop, D. J. Karmgard, K. Lannon, W. Li, N. Loukas, N. Marinelli, I. Mcalister, F. Meng, C. Mueller, Y. Musienko, M. Planer, R. Ruchti, P. Siddireddy, G. Smith, S. Taroni, M. Wayne, A. Wightman, M. Wolf, A. Woodard, J. Alimena, B. Bylsma, L. S. Durkin, S. Flowers, B. Francis, C. Hill, W. Ji, A. Lefeld, T. Y. Ling, B. L. Winer, S. Cooperstein, G. Dezoort, P. Elmer, J. Hardenbrook, N. Haubrich, S. Higginbotham, A. Kalogeropoulos, S. Kwan, D. Lange, M. T. Lucchini, J. Luo, D. Marlow, K. Mei, I. Ojalvo, J. Olsen, C. Palmer, P. Piroué, J. Salfeld-Nebgen, D. Stickland, C. Tully, Z. Wang, S. Malik, S. Norberg, A. Barker, V. E. Barnes, S. Das, L. Gutay, M. Jones, A. W. Jung, A. Khatiwada, B. Mahakud, D. H. Miller, G. Negro, N. Neumeister, C. C. Peng, S. Piperov, H. Qiu, J. F. Schulte, J. Sun, F. Wang, R. Xiao, W. Xie, T. Cheng, J. Dolen, N. Parashar, K. M. Ecklund, S. Freed, F. J. M. Geurts, M. Kilpatrick, A. Kumar, W. Li, B. P. Padley, R. Redjimi, J. Roberts, J. Rorie, W. Shi, A. G. Stahl Leiton, Z. Tu, A. Zhang, A. Bodek, P. de Barbaro, R. Demina, J. L. Dulemba, C. Fallon, T. Ferbel, M. Galanti, A. Garcia-Bellido, J. Han, O. Hindrichs, A. Khukhunaishvili, E. Ranken, P. Tan, R. Taus, B. Chiarito, J. P. Chou, A. Gandrakota, Y. Gershtein, E. Halkiadakis, A. Hart, M. Heindl, E. Hughes, S. Kaplan, S. Kyriacou, I. Laflotte, A. Lath, R. Montalvo, K. Nash, M. Osherson, H. Saka, S. Salur, S. Schnetzer, D. Sheffield, S. Somalwar, R. Stone, S. Thomas, P. Thomassen, H. Acharya, A. G. Delannoy, G. Riley, S. Spanier, O. Bouhali, A. Celik, M. Dalchenko, M. De Mattia, A. Delgado, S. Dildick, R. Eusebi, J. Gilmore, T. Huang, T. Kamon, S. Luo, D. Marley, R. Mueller, D. Overton, L. Perniè, D. Rathjens, A. Safonov, N. Akchurin, J. Damgov, F. De Guio, S. Kunori, K. Lamichhane, S. W. Lee, T. Mengke, S. Muthumuni, T. Peltola, S. Undleeb, I. Volobouev, Z. Wang, A. Whitbeck, S. Greene, A. Gurrola, R. Janjam, W. Johns, C. Maguire, A. Melo, H. Ni, K. Padeken, F. Romeo, P. Sheldon, S. Tuo, J. Velkovska, M. Verweij, M. W. Arenton, P. Barria, B. Cox, G. Cummings, R. Hirosky, M. Joyce, A. Ledovskoy, C. Neu, B. Tannenwald, Y. Wang, E. Wolfe, F. Xia, R. Harr, P. E. Karchin, N. Poudyal, J. Sturdy, P. Thapa, T. Bose, J. Buchanan, C. Caillol, D. Carlsmith, S. Dasu, I. De Bruyn, L. Dodd, F. Fiori, C. Galloni, B. Gomber, H. He, M. Herndon, A. Hervé, U. Hussain, P. Klabbers, A. Lanaro, A. Loeliger, K. Long, R. Loveless, J. Madhusudanan Sreekala, T. Ruggles, A. Savin, V. Sharma, W. H. Smith, D. Teague, S. Trembath-reichert, N. Woods, CMS Collaboration

**Affiliations:** 1 Yerevan Physics Institute, Yerevan, Armenia; 2 Institut für Hochenergiephysik, Wien, Austria; 3 Institute for Nuclear Problems, Minsk, Belarus; 4 Universiteit Antwerpen, Antwerp, Belgium; 5 Vrije Universiteit Brussel, Brussels, Belgium; 6 Université Libre de Bruxelles, Brussels, Belgium; 7 Ghent University, Ghent, Belgium; 8 Université Catholique de Louvain, Louvain-la-Neuve, Belgium; 9 Centro Brasileiro de Pesquisas Fisicas, Rio de Janeiro, Brazil; 10 Universidade do Estado do Rio de Janeiro, Rio de Janeiro, Brazil; 11 Universidade Estadual Paulista, Universidade Federal do ABC, São Paulo, Brazil; 12 Institute for Nuclear Research and Nuclear Energy, Bulgarian Academy of Sciences, Sofia, Bulgaria; 13 University of Sofia, Sofia, Bulgaria; 14 Beihang University, Beijing, China; 15 Institute of High Energy Physics, Beijing, China; 16 State Key Laboratory of Nuclear Physics and Technology, Peking University, Beijing, China; 17 Tsinghua University, Beijing, China; 18 Universidad de Los Andes, Bogotá, Colombia; 19 Universidad de Antioquia, Medellín, Colombia; 20 Faculty of Electrical Engineering, Mechanical Engineering and Naval Architecture, University of Split, Split, Croatia; 21 Faculty of Science, University of Split, Split, Croatia; 22 Institute Rudjer Boskovic, Zagreb, Croatia; 23 University of Cyprus, Nicosia, Cyprus; 24 Charles University, Prague, Czech Republic; 25 Escuela Politecnica Nacional, Quito, Ecuador; 26 Universidad San Francisco de Quito, Quito, Ecuador; 27 Academy of Scientific Research and Technology of the Arab Republic of Egypt, Egyptian Network of High Energy Physics, Cairo, Egypt; 28 National Institute of Chemical Physics and Biophysics, Tallinn, Estonia; 29 Department of Physics, University of Helsinki, Helsinki, Finland; 30 Helsinki Institute of Physics, Helsinki, Finland; 31 Lappeenranta University of Technology, Lappeenranta, Finland; 32 IRFU, CEA, Université Paris-Saclay, Gif-sur-Yvette, France; 33 Laboratoire Leprince-Ringuet, CNRS/IN2P3, Ecole Polytechnique, Institut Polytechnique de Paris, Paris, France; 34 Université de Strasbourg, CNRS, IPHC UMR 7178, Strasbourg, France; 35 Centre de Calcul de l’Institut National de Physique Nucleaire et de Physique des Particules, CNRS/IN2P3, Villeurbanne, France; 36 Université de Lyon, Université Claude Bernard Lyon 1, CNRS-IN2P3, Institut de Physique Nucléaire de Lyon, Villeurbanne, France; 37 Georgian Technical University, Tbilisi, Georgia; 38 Tbilisi State University, Tbilisi, Georgia; 39 RWTH Aachen University, I. Physikalisches Institut, Aachen, Germany; 40 RWTH Aachen University, III. Physikalisches Institut A, Aachen, Germany; 41 RWTH Aachen University, III. Physikalisches Institut B, Aachen, Germany; 42 Deutsches Elektronen-Synchrotron, Hamburg, Germany; 43 University of Hamburg, Hamburg, Germany; 44 Karlsruher Institut fuer Technologie, Karlsruhe, Germany; 45 Institute of Nuclear and Particle Physics (INPP), NCSR Demokritos, Aghia Paraskevi, Greece; 46 National and Kapodistrian University of Athens, Athens, Greece; 47 National Technical University of Athens, Athens, Greece; 48 University of Ioánnina, Ioannina, Greece; 49 MTA-ELTE Lendület CMS Particle and Nuclear Physics Group, Eötvös Loránd University, Budapest, Hungary; 50 Wigner Research Centre for Physics, Budapest, Hungary; 51 Institute of Nuclear Research ATOMKI, Debrecen, Hungary; 52 Institute of Physics, University of Debrecen, Debrecen, Hungary; 53 Eszterhazy Karoly University, Karoly Robert Campus, Gyongyos, Hungary; 54 Indian Institute of Science (IISc), Bangalore, India; 55 National Institute of Science Education and Research, HBNI, Bhubaneswar, India; 56 Panjab University, Chandigarh, India; 57 University of Delhi, Delhi, India; 58 Saha Institute of Nuclear Physics, HBNI, Kolkata, India; 59 Indian Institute of Technology Madras, Madras, India; 60 Bhabha Atomic Research Centre, Mumbai, India; 61 Tata Institute of Fundamental Research-A, Mumbai, India; 62 Tata Institute of Fundamental Research-B, Mumbai, India; 63 Indian Institute of Science Education and Research (IISER), Pune, India; 64 Institute for Research in Fundamental Sciences (IPM), Tehran, Iran; 65 University College Dublin, Dublin, Ireland; 66 INFN Sezione di Bari, Università di Bari, Politecnico di Bari, Bari, Italy; 67 INFN Sezione di Bologna, Università di Bologna, Bologna, Italy; 68 INFN Sezione di Catania, Università di Catania, Catania, Italy; 69 INFN Sezione di Firenze, Università di Firenze, Firenze, Italy; 70 INFN Laboratori Nazionali di Frascati, Frascati, Italy; 71 INFN Sezione di Genova, Università di Genova, Genoa, Italy; 72 INFN Sezione di Milano-Bicocca, Università di Milano-Bicocca, Milan, Italy; 73 INFN Sezione di Napoli, Università di Napoli ‘Federico II’ , Napoli, Italy, Università della Basilicata, Potenza, Italy, Università G. Marconi, Rome, Italy; 74 INFN Sezione di Padova, Università di Padova, Padova, Italy, Università di Trento, Trento, Italy; 75 INFN Sezione di Pavia, Università di Pavia, Pavia, Italy; 76 INFN Sezione di Perugia, Università di Perugia, Perugia, Italy; 77 INFN Sezione di Pisa, Università di Pisa, Scuola Normale Superiore di Pisa, Pisa, Italy; 78 INFN Sezione di Roma, Sapienza Università di Roma, Rome, Italy; 79 INFN Sezione di Torino, Università di Torino, Torino, Italy, Università del Piemonte Orientale, Novara, Italy; 80 INFN Sezione di Trieste, Università di Trieste, Trieste, Italy; 81 Kyungpook National University, Daegu, Korea; 82 Chonnam National University, Institute for Universe and Elementary Particles, Kwangju, Korea; 83 Hanyang University, Seoul, Korea; 84 Korea University, Seoul, Korea; 85 Department of Physics, Kyung Hee University, Seoul, Korea; 86 Sejong University, Seoul, Korea; 87 Seoul National University, Seoul, Korea; 88 University of Seoul, Seoul, Korea; 89 Sungkyunkwan University, Suwon, Korea; 90 Riga Technical University, Riga, Latvia; 91 Vilnius University, Vilnius, Lithuania; 92 National Centre for Particle Physics, Universiti Malaya, Kuala Lumpur, Malaysia; 93 Universidad de Sonora (UNISON), Hermosillo, Mexico; 94 Centro de Investigacion y de Estudios Avanzados del IPN, Mexico City, Mexico; 95 Universidad Iberoamericana, Mexico City, Mexico; 96 Benemerita Universidad Autonoma de Puebla, Puebla, Mexico; 97 Universidad Autónoma de San Luis Potosí, San Luis Potosí, Mexico; 98 University of Montenegro, Podgorica, Montenegro; 99 University of Auckland, Auckland, New Zealand; 100 University of Canterbury, Christchurch, New Zealand; 101 National Centre for Physics, Quaid-I-Azam University, Islamabad, Pakistan; 102 Faculty of Computer Science, Electronics and Telecommunications, AGH University of Science and Technology, Kraków, Poland; 103 National Centre for Nuclear Research, Swierk, Poland; 104 Institute of Experimental Physics, Faculty of Physics, University of Warsaw, Warsaw, Poland; 105 Laboratório de Instrumentação e Física Experimental de Partículas, Lisbon, Portugal; 106 Joint Institute for Nuclear Research, Dubna, Russia; 107 Petersburg Nuclear Physics Institute, Gatchina (St. Petersburg), Russia; 108 Institute for Nuclear Research, Moscow, Russia; 109 Institute for Theoretical and Experimental Physics named by A.I. Alikhanov of NRC ‘Kurchatov Institute’, Moscow, Russia; 110 Moscow Institute of Physics and Technology, Moscow, Russia; 111 National Research Nuclear University ‘Moscow Engineering Physics Institute’ (MEPhI), Moscow, Russia; 112 P.N. Lebedev Physical Institute, Moscow, Russia; 113 Skobeltsyn Institute of Nuclear Physics, Lomonosov Moscow State University, Moscow, Russia; 114 Novosibirsk State University (NSU), Novosibirsk, Russia; 115 Institute for High Energy Physics of National Research Centre ‘Kurchatov Institute’, Protvino, Russia; 116 National Research Tomsk Polytechnic University, Tomsk, Russia; 117 Tomsk State University, Tomsk, Russia; 118 Faculty of Physics and VINCA Institute of Nuclear Sciences, University of Belgrade, Belgrade, Serbia; 119 Centro de Investigaciones Energéticas Medioambientales y Tecnológicas (CIEMAT), Madrid, Spain; 120 Universidad Autónoma de Madrid, Madrid, Spain; 121 Universidad de Oviedo, Instituto Universitario de Ciencias y Tecnologías Espaciales de Asturias (ICTEA), Oviedo, Spain; 122 Instituto de Física de Cantabria (IFCA), CSIC-Universidad de Cantabria, Santander, Spain; 123 University of Colombo, Colombo, Sri Lanka; 124 Department of Physics, University of Ruhuna, Matara, Sri Lanka; 125 CERN, European Organization for Nuclear Research, Geneva, Switzerland; 126 Paul Scherrer Institut, Villigen, Switzerland; 127 ETH Zurich-Institute for Particle Physics and Astrophysics (IPA), Zurich, Switzerland; 128 Universität Zürich, Zurich, Switzerland; 129 National Central University, Chung-Li, Taiwan; 130 National Taiwan University (NTU), Taipei, Taiwan; 131 Department of Physics, Faculty of Science, Chulalongkorn University, Bangkok, Thailand; 132 Physics Department, Science and Art Faculty, Çukurova University, Adana, Turkey; 133 Physics Department, Middle East Technical University, Ankara, Turkey; 134 Bogazici University, Istanbul, Turkey; 135 Istanbul Technical University, Istanbul, Turkey; 136 Istanbul University, Istanbul, Turkey; 137 Institute for Scintillation Materials of National Academy of Science of Ukraine, Kharkov, Ukraine; 138 National Scientific Center, Kharkov Institute of Physics and Technology, Kharkov, Ukraine; 139 University of Bristol, Bristol, UK; 140 Rutherford Appleton Laboratory, Didcot, UK; 141 Imperial College, London, UK; 142 Brunel University, Uxbridge, UK; 143 Baylor University, Waco, USA; 144 Catholic University of America, Washington DC, USA; 145 The University of Alabama, Tuscaloosa, USA; 146 Boston University, Boston, USA; 147 Brown University, Providence, USA; 148 University of California, Davis, Davis, USA; 149 University of California, Los Angeles, USA; 150 University of California, Riverside, Riverside, USA; 151 University of California, San Diego, La Jolla, USA; 152 Department of Physics, University of California, Santa Barbara, Santa Barbara, USA; 153 California Institute of Technology, Pasadena, USA; 154 Carnegie Mellon University, Pittsburgh, USA; 155 University of Colorado Boulder, Boulder, USA; 156 Cornell University, Ithaca, USA; 157 Fermi National Accelerator Laboratory, Batavia, USA; 158 University of Florida, Gainesville, USA; 159 Florida International University, Miami, USA; 160 Florida State University, Tallahassee, USA; 161 Florida Institute of Technology, Melbourne, USA; 162 University of Illinois at Chicago (UIC), Chicago, USA; 163 The University of Iowa, Iowa City, USA; 164 Johns Hopkins University, Baltimore, USA; 165 The University of Kansas, Lawrence, USA; 166 Kansas State University, Manhattan, USA; 167 Lawrence Livermore National Laboratory, Livermore, USA; 168 University of Maryland, College Park, USA; 169 Massachusetts Institute of Technology, Cambridge, USA; 170 University of Minnesota, Minneapolis, USA; 171 University of Mississippi, Oxford, USA; 172 University of Nebraska-Lincoln, Lincoln, USA; 173 State University of New York at Buffalo, Buffalo, USA; 174 Northeastern University, Boston, USA; 175 Northwestern University, Evanston, USA; 176 University of Notre Dame, Notre Dame, USA; 177 The Ohio State University, Columbus, USA; 178 Princeton University, Princeton, USA; 179 University of Puerto Rico, Mayagüez, USA; 180 Purdue University, West Lafayette, USA; 181 Purdue University Northwest, Hammond, USA; 182 Rice University, Houston, USA; 183 University of Rochester, Rochester, USA; 184 Rutgers, The State University of New Jersey, Piscataway, USA; 185 University of Tennessee, Knoxville, USA; 186 Texas A&M University, College Station, USA; 187 Texas Tech University, Lubbock, USA; 188 Vanderbilt University, Nashville, USA; 189 University of Virginia, Charlottesville, USA; 190 Wayne State University, Detroit, USA; 191 University of Wisconsin-Madison, Madison, WI USA; 192 CERN, 1211 Geneva 23, Switzerland

## Abstract

Anisotropies in the initial energy density distribution of the quark-gluon plasma created in high energy heavy ion collisions lead to anisotropies in the azimuthal distributions of the final-state particles known as collective anisotropic flow. Fourier harmonic decomposition is used to quantify these anisotropies. The higher-order harmonics can be induced by the same order anisotropies (linear response) or by the combined influence of several lower order anisotropies (nonlinear response) in the initial state. The mixed higher-order anisotropic flow and nonlinear response coefficients of charged particles are measured as functions of transverse momentum and centrality in $\mathrm {PbPb}$ collisions at nucleon-nucleon center-of-mass energies $\sqrt{\smash [b]{s_{_{\mathrm {NN}}}}} = 2.76$ and 5.02$\,\text {TeV}$ with the CMS detector. The results are compared with viscous hydrodynamic calculations using several different initial conditions, as well as microscopic transport model calculations. None of the models provides a simultaneous description of the mixed higher-order flow harmonics and nonlinear response coefficients.

## Introduction

The azimuthal anisotropy of particle production in a heavy ion collision can be characterized by the Fourier expansion of the particle azimuthal angle distribution [1],

1 $$\begin{aligned} \frac{dN}{d\phi }=\frac{N}{2\pi }\sum _{n=-\infty }^{+\infty }V_n e^{-in\phi }, \end{aligned}$$

where $V_n=v_n\exp (in\varPsi _n)$ is the *n*th complex anisotropic flow coefficient [2]. The $v_n$ and $\varPsi _n$ are the magnitude and phase (also known as the *n*th order symmetry plane angle) of $V_n$, respectively. Anisotropic flow plays a major role in probing the properties of the produced medium in heavy ion collisions at the BNL RHIC [3–6] and CERN LHC [7–9]. Studies of flow harmonics higher than the second order [10–12], flow fluctuations [13–16], the correlation between the magnitude and phase of different harmonics [17–24], and the transverse momentum ($p_{\mathrm {T}}$) and pseudorapidity $(\eta )$ dependence of symmetry plane angles [25, 26], have led to a broader and deeper understanding of the initial conditions [3, 27] and the properties of the produced hot and dense matter. There are significant correlations between the symmetry plane angles of different orders [20], which indicate that higher-order mixed harmonics can be studied with respect to multiple lower-order symmetry plane angles.

In hydrodynamical models describing the quark-gluon plasma (QGP) created in relativistic heavy ion collisions, anisotropic flow arises from the evolution of the medium in the presence of an anisotropy in the initial-state energy density, as characterized by the eccentricities $\epsilon _n$ [10]. The magnitudes of the second- and third-order harmonic final state coefficients, $v_{2}$ and $v_{3}$, are to a good approximation linearly proportional to the initial-state anisotropies, $\epsilon _{2}$ and $\epsilon _{3}$, respectively [10, 17]. In contrast, $V_4$ and higher harmonics can arise from initial-state anisotropies in the same-order harmonic (linear response) or can be induced by lower-order harmonics (nonlinear response) [1, 28, 29]. More specifically, these harmonics can be decomposed into linear and nonlinear response contributions as follows [1, 28]:

2 $$\begin{aligned} \begin{aligned} V_4&= V_{4 L} + \chi _{422} V_2^2,\\ V_5&= V_{5 L} + \chi _{523} V_2 V_3,\\ V_6&= V_{6 L} + \chi _{624} V_2 V_{4L} + \chi _{633} V_3^2 + \chi _{6222} V_2^3,\\ V_7&= V_{7 L} + \chi _{725} V_2 V_{5L} + \chi _{734} V_3 V_{4L} + \chi _{7223} V_2^2 V_3, \end{aligned} \end{aligned}$$

where $V_{nL}$ denotes the part of $V_n$ that is not induced by lower-order harmonics [29–31], and the $\chi$ are the nonlinear response coefficients. Each nonlinear response coefficient has its associated mixed harmonic, which is $V_n$ measured with respect to the lower-order symmetry plane angle or angles. The strength of each nonlinear response coefficient determines the magnitude of its associated mixed harmonic. The $V_1$ terms are neglected in the decomposition in Eq. (2) because the correlation between $V_{n}$ and $V_{1}V_{n-1}$ was shown to be negligible after correcting $V_{1}$ for global momentum conservation [28]. This analysis focuses on the terms that only involve the two largest anisotropic flow coefficients $V_2$ and $V_3$ on the right-hand side of Eq. (2). The procedures used to extract both mixed-harmonic and nonlinear response coefficients are given in Sect. 4.

It is difficult to use measured $v_2$ and $v_3$ coefficients to evaluate hydrodynamic theories because these flow observables have a strong dependence on the initial anisotropies, which cannot be experimentally determined or tightly constrained. In contrast, most of the nonlinear response coefficients are not strongly sensitive to the initial anisotropies, which largely cancel in the dimensionless ratios used to determine these coefficients [1, 28, 31, 32]. As a result, their experimental values can serve as unique and robust probes of hydrodynamic behavior of the QGP [31].

Most previous flow measurements focused on $V_n$ (overall flow), i.e., $v_n$ with respect to $\varPsi _n$, which does not separate the linear and nonlinear parts of Eq. (2). Direct measurements of the mixed higher-order flow harmonics, $v_4$ and $v_6$ with respect to $\varPsi _2$, already exist at both RHIC [33] and LHC [11] energies, but were performed using the event plane method [34]. This method has been criticized for yielding an ambiguous measure lying somewhere between the event-averaged mean value $\left\langle v_n \right\rangle$ and the root-mean-square value $\sqrt{\left\langle {v_n^2} \right\rangle }$ of the $v_n$ distribution, depending on the resolution of the method [13, 16, 35]. This ambiguity can be removed by using the scalar-product method [35, 36], which always measures the root-mean-square values of $v_n$. The difference between the two methods is typically a few percent for $v_2$, $\sim$ 10% for $v_3$, and much larger for mixed harmonics [35].

This paper presents the mixed higher-order flow harmonics and nonlinear response coefficients for $n = 4$, 5, 6, and 7 using the scalar-product method. These variables are measured in $\mathrm {PbPb}$ collisions at nucleon-nucleon center-of-mass energies $\sqrt{\smash [b]{s_{_{\mathrm {NN}}}}} = 2.76$ and 5.02$\,\text {TeV}$, as functions of collision centrality and charged particle $p_{\mathrm {T}}$ in the region $|\eta |<0.8$. To compare the mixed flow harmonics with the overall flow coefficients, the higher-order flow harmonics with respect to the same-order symmetry plane, measured using the scalar-product method, are also presented.

## The CMS detector

The central feature of the CMS apparatus is a superconducting solenoid of 6$\, \text {m}$ internal diameter, providing a nearly constant magnetic field of 3.8$\, \text {T}$. Within the solenoid volume are a silicon pixel and strip tracker, a lead tungstate crystal electromagnetic calorimeter, and a brass and scintillator hadron calorimeter, each composed of a barrel and two endcap sections. In this analysis, the tracker and the forward hadron (HF) calorimeter subsystems are of particular importance. The HF uses steel as an absorber and quartz fibers as the sensitive material. The two halves of the HF are located 11.2$\, \text {m}$ from the center of the interaction region, one on each end, and together they provide coverage in the range $3.0< |\eta | < 5.2$. These calorimeters are azimuthally subdivided into $20^{\circ }$ modular wedges and further segmented to form $0.175{\times }0.175$ $(\varDelta \eta {\times }\varDelta \phi )$ “towers”, where the angle $\phi$ is in radians. The silicon tracker measures charged particles within the range $|\eta | < 2.5$. It consists of 1440 silicon pixel and 15,148 silicon strip detector modules. For nonisolated particles of $1< p_{\mathrm {T}} < 10{\,\text {GeV/}c}$ and $|\eta | < 1.4$, the track resolutions are typically 1.5% in $p_{\mathrm {T}}$ and 25–90 (45–150)$\,\upmu \text {m}$ in the transverse (longitudinal) impact parameter [37]. The Beam Pick-up Timing for the eXperiments (BPTX) devices are located around the beam pipe at a distance of 175$\, \text {m}$ from the interaction region on both sides, and are designed to provide precise information on the LHC bunch structure and timing of the incoming beams. A more detailed description of the CMS detector, together with a definition of the coordinate system used and the relevant kinematic variables, can be found in Ref. [38]. The Monte Carlo simulation of the particle propagation and detector response is based on the Geant4 [39] program.

## Event and track selections

This analysis is performed using minimum bias $\mathrm {PbPb}$ data collected with the CMS detector at $\sqrt{\smash [b]{s_{_{\mathrm {NN}}}}} =$ 5.02 and 2.76$\,\text {TeV}$ in 2015 and 2011, corresponding to integrated luminosities of 13$\,\mu \text {b}^{-1}$ and 3.9$\,\mu \text {b}^{-1}$, respectively. The minimum bias trigger [40] used in this analysis requires coincident signals in the HF calorimeters at both ends of the CMS detector with total energy deposits above a predefined energy threshold of approximately 1$\,\text {GeV}$ and the presence of both colliding bunches in the interaction region as determined using the BPTX. By requiring colliding bunches, events due to noise (e.g., cosmic rays and beam backgrounds) are largely suppressed. In the offline analysis, events are required to have at least one reconstructed primary vertex, which is chosen as the reconstructed vertex with the largest number of associated tracks. The primary vertex is formed by two or more associated tracks and is required to have a distance of less than 15$\, \text {cm}$ along the beam axis from the center of the nominal interaction region and less than 0.15$\, \text {cm}$ from the beam position in the transverse plane. An additional selection of hadronic collisions is applied by requiring at least three towers, each with total energy above 3$\,\text {GeV}$ in each of the two HF calorimeters. The average number of collisions per bunch crossing is less than 0.001 for the events used in this analysis, with a pileup fraction less than 0.05%, which has a negligible effect on the results. Events are classified using a centrality variable that is related to the degree of geometric overlap between the two colliding nuclei. Events with complete (no) overlap are denoted as centrality 0 (100)%, where the number is the fraction of events in a given class with respect to the total number of inelastic hadronic collisions. The centrality is determined offline via the sum of the HF energies in each event. Very central events (centrality approaching 0%) are characterized by a large energy deposit in the HF calorimeters. The results reported in this paper are presented up to 60% in centrality. The minimum bias trigger and event selections are fully efficient in this centrality range.

Track reconstruction [37, 41] is performed in two iterations to ease the computational load for high-multiplicity central $\mathrm {PbPb}$ collisions. The first iteration reconstructs tracks from signals (“hits”) in the silicon pixel and strip detectors compatible with a trajectory of $p_{\mathrm {T}} >0.9{\,\text {GeV/}c}$. The significance of the separation along the beam axis (*z*) between the track and the primary vertex, $d_z/\sigma (d_z)$, and the significance of the impact parameter relative to the primary vertex transverse to the beam, $d_{\mathrm {0}}/\sigma (d_{\mathrm {0}})$, must be less than 2. In addition, the relative uncertainty of the $p_{\mathrm {T}}$ measurement, $\sigma (p_{\mathrm {T}})/$ $p_{\mathrm {T}}$, must be less than 5%, and tracks are required to have at least 11 out of the possible 14 hits along their trajectories in the pixel and strip trackers. To reduce the number of misidentified tracks, the chi-squared per degree of freedom, $\chi ^2/\mathrm {dof}$, associated with fitting the track trajectory through the different pixel and strip layers, must be less than 0.15 times the total number of layers having hits along the trajectory of the track. The second iteration reconstructs tracks compatible with a trajectory of $p_{\mathrm {T}} >0.2{\,\text {GeV/}c}$ using solely the pixel detector. These tracks are required to have $d_{z}/\sigma (d_{z}) < 6$ and a fit $\chi ^2/\mathrm {dof}$ value less than 9 times the number of layers with hits along the trajectory of the track. In the final analysis, first iteration tracks with $p_{\mathrm {T}} > 1.0{\,\text {GeV/}c}$ are combined with pixel-detector-only tracks that have $0.2< p_{\mathrm {T}} < 2.4{\,\text {GeV/}c}$. After removing duplicates [7], the merged track collection has a combined geometric acceptance and efficiency exceeding 60% for $p_{\mathrm {T}}$ $\approx 1.0{\,\text {GeV/}c}$ and $|\eta |<0.8$, as determined using the hydjet event generator [42]. When the track $p_{\mathrm {T}}$ is below 1${\,\text {GeV/}c}$, the acceptance and efficiency steadily drops, reaching approximately 40% at $p_{\mathrm {T}} \approx 0.3{\,\text {GeV/}c}$, which is the lower limit for $p_{\mathrm {T}}$ in this analysis.

## Analysis technique

The analysis technique follows the method described in Refs. [1, 28] using detector information from both HF and the tracker. The notation $V_n=v_n\exp (in\varPsi _n)=\left\langle e^{in\phi } \right\rangle$ in Eq. (1) will be replaced by the measured complex flow vector $Q_n$ with real and imaginary parts defined as

3 $$\begin{aligned} {\text {Re}}(Q_n)= & {} \frac{1}{\sum {w_j}}\sum \limits _j^M {w_j}\cos \left( {n{\phi _j}} \right) \nonumber \\&- \left\langle \frac{1}{\sum {w_j}}\sum \limits _j^M {w_j}\cos \left( {n{\phi _j}} \right) \right\rangle , \end{aligned}$$

4 $$\begin{aligned} {\text {Im}}(Q_n)= & {} \frac{1}{\sum {w_j}}\sum \limits _j^M {w_j}\sin \left( {n{\phi _j}} \right) \nonumber \\&- \left\langle \frac{1}{\sum {w_j}}\sum \limits _j^M {w_j}\sin \left( {n{\phi _j}} \right) \right\rangle , \end{aligned}$$

where *M* represents the number of tracks or HF towers used for calculating the *Q* vector, $\phi _j$ is the azimuthal angle of the *j*th track or HF tower, and $w_j$ is a weighting factor equal to transverse energy for HF *Q* vectors. To correct for the tracking inefficiency, $w_j = 1/\varepsilon _j$ is the inverse of the tracking efficiency $\varepsilon _j(p_{\mathrm {T}}, \eta )$ of the *j*th track. Unlike the averages over particles in a single event in the definitions of $Q_n$, the angle brackets in Eqs. (3) and (4) denote an average over all the events within a given centrality range. Subtraction of the event-averaged quantity removes biases due to the detector acceptance.

The mixed higher-order harmonics in each $p_{\mathrm {T}}$ range are extracted using the scalar-product method as shown in Eqs. (5)–(9) [1], which describe the various harmonics measured with respect to symmetry plane angles of different orders. Equations (5)–(9) show $v_4$ with respect to the second-order, $v_5$ with respect to the second- and third-order, $v_6$ with respect to the second-order, $v_6$ with respect to the third-order, and $v_7$ with respect to the second- and third-order symmetry plane angles, respectively.

5 $$\begin{aligned} v_{4}\{\varPsi _{22}\}\equiv & {} \frac{{\mathrm {Re}}\langle Q_{4} Q_{2A}^{*}Q_{2B}^{*}\rangle }{\sqrt{{\mathrm {Re}}\langle Q_{2A}Q_{2A}Q_{2B}^{*}Q_{2B}^{*} \rangle }} \end{aligned}$$

6 $$\begin{aligned} v_{5}\{\varPsi _{23}\}\equiv & {} \frac{{\mathrm {Re}}\langle Q_{5} Q_{2A}^{*}Q_{3B}^{*}\rangle }{\sqrt{{\mathrm {Re}}\langle Q_{2A}Q_{3A}Q_{2B}^{*}Q_{3B}^{*} \rangle }} \end{aligned}$$

7 $$\begin{aligned} v_{6}\{\varPsi _{222}\}\equiv & {} \frac{{\mathrm {Re}}\langle Q_{6} Q_{2A}^{*}Q_{2B}^{*}Q_{2B}^{*}\rangle }{\sqrt{{\mathrm {Re}}\langle Q_{2A}Q_{2A}Q_{2A}Q_{2B}^{*}Q_{2B}^{*}Q_{2B}^{*} \rangle }} \end{aligned}$$

8 $$\begin{aligned} v_{6}\{\varPsi _{33}\}\equiv & {} \frac{{\mathrm {Re}}\langle Q_{6} Q_{3A}^{*}Q_{3B}^{*}\rangle }{\sqrt{{\mathrm {Re}}\langle Q_{3A}Q_{3A}Q_{3B}^{*}Q_{3B}^{*} \rangle }} \end{aligned}$$

9 $$\begin{aligned} v_{7}\{\varPsi _{223}\}\equiv & {} \frac{{\mathrm {Re}}\langle Q_{7} Q_{2A}^{*}Q_{2B}^{*}Q_{3B}^{*}\rangle }{\sqrt{{\mathrm {Re}}\langle Q_{2A}Q_{2A}Q_{3A}Q_{2B}^{*}Q_{2B}^{*}Q_{3B}^{*} \rangle }} \end{aligned}$$

Here, $Q_{nA}$ and $Q_{nB}$ are vectors from two different parts of the detector, specifically the positive and negative sides of HF, $Q_{n}$ is the vector from charged particles in each $p_{\mathrm {T}}$ range within $|\eta |<0.8$, and angle brackets denote the average (weighted by the number of particles) over all events within a given centrality range. The minimum $\eta$ gap between tracks used to find the charged-particle *Q* vector and towers used for the HF *Q* vectors is 2.2 units of $\eta$.

With the assumption that the linear and nonlinear terms in Eq. (2) are uncorrelated, the nonlinear response coefficients in each $p_{\mathrm {T}}$ range can be expressed as [1, 28],

10 $$\begin{aligned} \chi _{422}= & {} \frac{{\mathrm {Re}}\langle Q_{4} Q_{2A}^{*}Q_{2B}^{*}\rangle }{{{\mathrm {Re}}\langle Q_{2}Q_{2}Q_{2A}^{*}Q_{2B}^{*} \rangle }}, \end{aligned}$$

11 $$\begin{aligned} \chi _{523}= & {} \frac{{\mathrm {Re}}\langle Q_{5} Q_{2A}^{*}Q_{3B}^{*}\rangle }{{{\mathrm {Re}}\langle Q_{2}Q_{3}Q_{2A}^{*}Q_{3B}^{*} \rangle }}, \end{aligned}$$

12 $$\begin{aligned} \chi _{6222}= & {} \frac{{\mathrm {Re}}\langle Q_{6} Q_{2A}^{*}Q_{2B}^{*}Q_{2B}^{*}\rangle }{{{\mathrm {Re}}\langle Q_{2}Q_{2}Q_{2}Q_{2A}^{*}Q_{2B}^{*}Q_{2B}^{*} \rangle }}, \end{aligned}$$

13 $$\begin{aligned} \chi _{633}= & {} \frac{{\mathrm {Re}}\langle Q_{6} Q_{3A}^{*}Q_{3B}^{*}\rangle }{{{\mathrm {Re}}\langle Q_{3}Q_{3}Q_{3A}^{*}Q_{3B}^{*} \rangle }}, \end{aligned}$$

14 $$\begin{aligned} \chi _{7223}= & {} \frac{{\mathrm {Re}}\langle Q_{7} Q_{2A}^{*}Q_{2B}^{*}Q_{3B}^{*}\rangle }{{{\mathrm {Re}}\langle Q_{2}Q_{2}Q_{3}Q_{2A}^{*}Q_{2B}^{*}Q_{3B}^{*} \rangle }}, \end{aligned}$$

where the charged-particle $Q_{n}$ vector enters both the numerator and the denominator.

## Systematic uncertainties

Six sources of systematic uncertainties are considered in this analysis. The systematic uncertainty due to vertex position selection is estimated by comparing the results with events from vertex position ranges $|v_z |<3$ $\, \text {cm}$ to $3<|v_z |<15$ $\, \text {cm}$. For both mixed harmonic and nonlinear response coefficients, this uncertainty is estimated to be 1–3%, with no dependence on $p_{\mathrm {T}}$ or centrality. Systematic uncertainty due to track quality requirements are examined by varying the track selections for $d_z/\sigma (d_z)$ and $d_{\mathrm {0}}/\sigma (d_{\mathrm {0}})$ from 1.5 to 5, the pixel track $d_{z}/\sigma (d_{z})$ from 5 to 10, and the fit $\chi ^2/\mathrm {dof}$ value from 7 to 18 times the number of layers with hits. The uncertainty is estimated to be 1–4% depending on $p_{\mathrm {T}}$ and centrality for both mixed harmonic and nonlinear response coefficients.

The charged-particle tracking efficiency depends on the efficiency of detecting different types of charged particles and the species composition of the set of particles. Two event generators (hydjet [42] and epos lhc [43]) with different particle composition are used to study the tracking efficiency, and the systematic uncertainty is obtained by comparing the results using efficiencies from the two generators mentioned above. The systematic uncertainty from this source is 3% for the mixed harmonics and less than 1% for the nonlinear response coefficients, with no dependence on $p_{\mathrm {T}}$ or centrality.

The sensitivity of the results to the centrality calibration is evaluated by varying the trigger and event selection efficiency by $\pm 2$%. The resulting uncertainty is estimated to be less than 1%. The minimum $\eta$ gap between the correlated charged particles and the *Q* vectors in the HF region is changed from 2.2 to 3.2 units of $\eta$ (achieved by changing the $\eta$ ranges of the HF *Q* vectors) to estimate the uncertainty due to short-range correlations from resonance decays and jets. This study results in a systematic uncertainty of 1–8%, depending on both $p_{\mathrm {T}}$ and centrality. This $\eta$ gap uncertainty also includes a possible physics effect from the $\eta$-dependent fluctuations of symmetry plane angles [26, 44], although a recent study from the ALICE experiment indicates that this effect is small for correlations between symmetry plane angles of different order [45].

When the same set of HF towers are used for different *Q* vectors in the equations of mixed harmonic and nonlinear response coefficients, the product of these *Q* vectors contains self-correlations. An algorithm for removing the duplicated terms when multiplying two or more *Q* vectors, the same as the approach of Ref. [46], is used. The algorithm only works perfectly when the detector has fine granularity and there is no merging of HF towers. Therefore, the difference before and after correcting for this effect is taken as the systematic uncertainty, yielding values which depend on centrality but are always less than 3%.

The different systematic sources described above are added in quadrature to obtain the overall systematic uncertainty, which is about 10% at low $p_{\mathrm {T}}$ and decreases to around 5% for $p_{\mathrm {T}}$ larger than 1${\,\text {GeV/}c}$. As a function of centrality, the overall systematic uncertainty ranges from 3 to 9% for different coefficients, with larger uncertainties for central events.

## Results

**Fig. 1:**
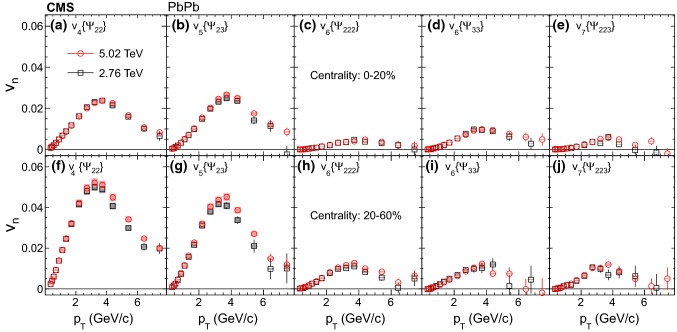
Mixed higher-order flow harmonics, $v_4\{\varPsi _{22}\}$, $v_5\{\varPsi _{23}\}$, $v_6\{\varPsi _{222}\}$, $v_6\{\varPsi _{33}\}$, and $v_7\{\varPsi _{223}\}$ from the scalar-product method at $\sqrt{\smash [b]{s_{_{\mathrm {NN}}}}} = 2.76$ and 5.02$\,\text {TeV}$ as a function of $p_{\mathrm {T}}$ in the 0–20% (upper row) and 20–60% (lower row) centrality ranges. Statistical (bars) and systematic (shaded boxes) uncertainties are shown

The measurements in this paper are presented using tracks in the range of $|\eta | < 0.8$. Figure 1 shows the mixed higher-order flow harmonics, $v_4\{\varPsi _{22}\}$, $v_5\{\varPsi _{23}\}$, $v_6\{\varPsi _{222}\}$, $v_6\{\varPsi _{33}\}$, and $v_7\{\varPsi _{223}\}$ from the scalar-product method at $\sqrt{\smash [b]{s_{_{\mathrm {NN}}}}} = 2.76$ and 5.02$\,\text {TeV}$ as a function of $p_{\mathrm {T}}$ in the 0–20% (upper row) and 20–60% (lower row) centrality ranges.

**Fig. 2:**
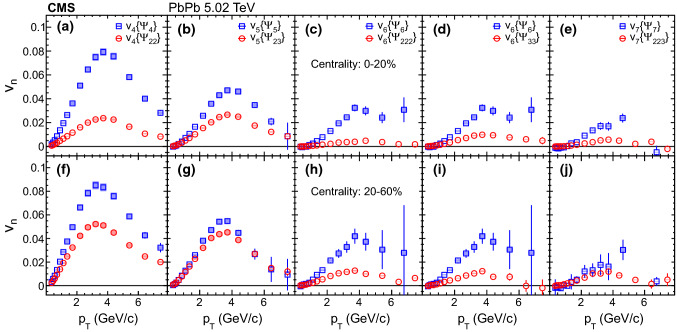
Comparison of mixed higher-order flow harmonics, $v_4\{\varPsi _{22}\}$, $v_5\{\varPsi _{23}\}$, $v_6\{\varPsi _{222}\}$, $v_6\{\varPsi _{33}\}$ and $v_7\{\varPsi _{223}\}$ with the corresponding overall flow, $v_4\{\varPsi _{4}\}$, $v_5\{\varPsi _{5}\}$, $v_6\{\varPsi _{6}\}$, $v_6\{\varPsi _{6}\}$ and $v_7\{\varPsi _{7}\}$, respectively, at $\sqrt{\smash [b]{s_{_{\mathrm {NN}}}}} = 5.02\,\text {TeV}$ as a function $p_{\mathrm {T}}$ in the 0–20% (upper row) and 20–60% (lower row) centrality ranges. Statistical (bars) and systematic (shaded boxes) uncertainties are shown

It is observed that the shapes of the mixed higher-order flow harmonics as a function of $p_{\mathrm {T}}$ are qualitatively similar to the published overall flow harmonics with respect to $\varPsi _n$ [7, 11], first increasing at low $p_{\mathrm {T}}$, reaching a maximum at about 3–4${\,\text {GeV/}c}$, then decreasing at higher $p_{\mathrm {T}}$. This may indicate that, for each $p_{\mathrm {T}}$ region, the underlying physics processes that generate the flow harmonics are the same for the nonlinear and the linear parts. Similar to previous observation that the overall flow shows a weak energy dependence from RHIC to LHC energies [7, 8], the mixed harmonics are also found to be consistent between the two collision energies within the uncertainties, except for $v_4\{\varPsi _{22}\}$ and $v_5\{\varPsi _{23}\}$ at $p_{\mathrm {T}}$ larger than 3${\,\text {GeV/}c}$ in the mid-central collisions, with 5.02$\,\text {TeV}$ results slightly above 2.76$\,\text {TeV}$ results.

**Fig. 3:**
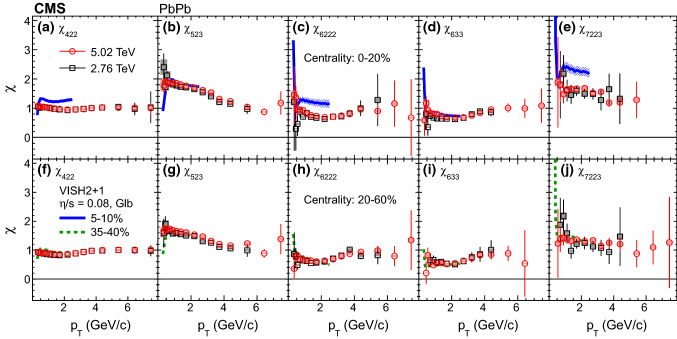
Nonlinear response coefficients, $\chi _{422}$, $\chi _{523}$, $\chi _{6222}$, $\chi _{633}$, and $\chi _{7223}$ from the scalar-product method at $\sqrt{\smash [b]{s_{_{\mathrm {NN}}}}} = 2.76$ and 5.02$\,\text {TeV}$ as a function of $p_{\mathrm {T}}$ in the 0–20% (upper row) and 20–60% (lower row) centrality ranges. Statistical (bars) and systematic (shaded boxes) uncertainties are shown. The results are compared with hydrodynamic predictions [30] at $\sqrt{\smash [b]{s_{_{\mathrm {NN}}}}} = 2.76\,\text {TeV}$ with $\eta /s = 0.08$ and Glauber initial conditions in the 5–10% (blue lines) and 35–40% (dashed green lines) centrality ranges

A direct comparison of the mixed higher-order flow harmonics and overall flow at 5.02$\,\text {TeV}$ is presented in Fig. 2 as a function of $p_{\mathrm {T}}$ in the two centrality ranges. Hydrodynamic models predict that the contribution of the nonlinear response to the overall flow increases towards peripheral collisions for $v_4$ and $v_5$ [17, 29, 47]. From a comparison of the relative contribution in the two centrality ranges, the present results are consistent with these predictions, as well as an estimate by the ATLAS Collaboration using a two-component fit of the correlation between flow harmonics [21], and a recent study of the nonlinear mode by the ALICE Collaboration [45]. By comparing different harmonics, the contribution of the nonlinear response for $v_5$ is larger than those for the other harmonics in the centrality range 20–60%.

The nonlinear response coefficients, $\chi _{422}$, $\chi _{523}$, $\chi _{6222}$, $\chi _{633}$, and $\chi _{7223}$ are presented in Fig. 3 as a function of $p_{\mathrm {T}}$ in the two centrality ranges. It is observed that the odd harmonic coefficients $\chi _{523}$ and $\chi _{7223}$ are larger than those for the even harmonics for $p_{\mathrm {T}}$ less than 3${\,\text {GeV/}c}$ in the two explored centrality ranges. The values for the even harmonics first decrease slightly as $p_{\mathrm {T}}$ increases, reach a minimum at $p_{\mathrm {T}}$ about 2${\,\text {GeV/}c}$, and then slowly increase until appearing to plateau for $p_{\mathrm {T}}$ above 4${\,\text {GeV/}c}$. The results are compared with viscous hydrodynamic predictions [30] at $\sqrt{\smash [b]{s_{_{\mathrm {NN}}}}} = 2.76\,\text {TeV}$ with $\eta /s = 0.08$ (where $\eta /s$ is the shear viscosity to entropy density ratio of the hydrodynamic medium, and here $\eta$ denotes shear viscosity rather than pseudorapidity) and Glauber initial conditions in two centrality ranges (5–10% and 35–40%) which roughly match those of the data (0–20% and 20–60%). In the model, as $p_{\mathrm {T}}$ increases from 0.3 to 1${\,\text {GeV/}c}$, the predicted coefficients increase for $n = 4$ and 5, but decrease and then increase for $n = 6$ and 7, with a much stronger $p_{\mathrm {T}}$ dependence than the data. The strong $p_{\mathrm {T}}$ dependence, attributed to the large variance of the flow angles $\varPsi _n$ at small $p_{\mathrm {T}}$ [30], is not observed in data for $n = 4$ and 5.

**Fig. 4:**
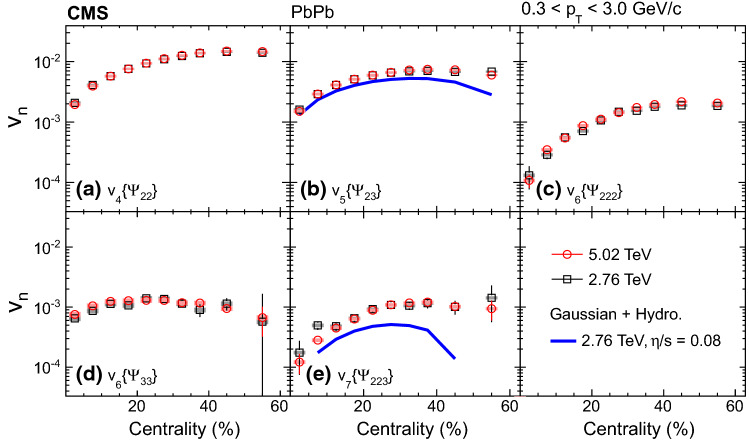
Mixed higher-order flow harmonics, $v_4\{\varPsi _{22}\}$, $v_5\{\varPsi _{23}\}$, $v_6\{\varPsi _{222}\}$, $v_6\{\varPsi _{33}\}$, and $v_7\{\varPsi _{223}\}$ from the scalar-product method at $\sqrt{\smash [b]{s_{_{\mathrm {NN}}}}} = 2.76$ and 5.02$\,\text {TeV}$, as a function of centrality. Statistical (bars) and systematic (shaded boxes) uncertainties are shown. Hydrodynamic predictions [1] with $\eta /s = 0.08$ (blue lines) at 2.76$\,\text {TeV}$ are shown in (**b**) and (**e**)

Figure 4 shows the mixed higher-order flow harmonics, $v_4\{\varPsi _{22}\}$, $v_5\{\varPsi _{23}\}$, $v_6\{\varPsi _{222}\}$, $v_6\{\varPsi _{33}\}$, and $v_7\{\varPsi _{223}\}$ from the scalar-product method, as a function of centrality in the $p_{\mathrm {T}}$ range from 0.3 to 3.0${\,\text {GeV/}c}$. Hydrodynamic predictions with a deformed symmetric Gaussian density profile as the initial conditions for $v_5\{\varPsi _{23}\}$ and $v_7\{\varPsi _{223}\}$ [1] at $\sqrt{\smash [b]{s_{_{\mathrm {NN}}}}} = 2.76\,\text {TeV}$ are compared with the data. The model qualitatively describes $v_5\{\varPsi _{23}\}$ in the 0–40% centrality range but underestimates the result for more peripheral collisions. For $v_7\{\varPsi _{223}\}$, the predicted values are much smaller than the data, especially for centrality from 35 to 50%.

**Fig. 5:**
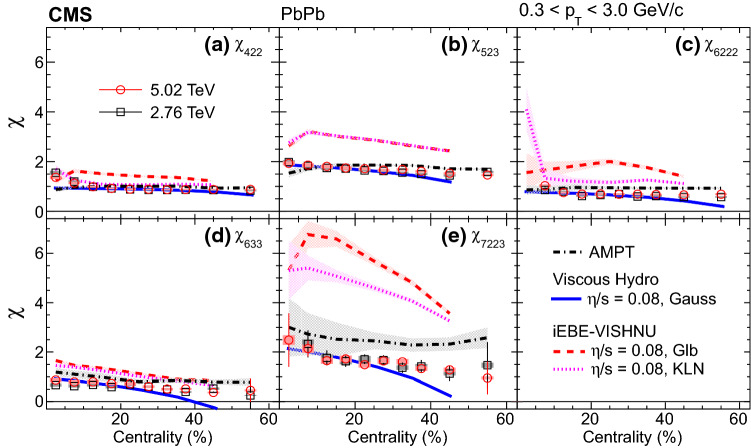
Nonlinear response coefficients, $\chi _{422}$, $\chi _{523}$, $\chi _{6222}$, $\chi _{633}$, and $\chi _{7223}$ from the scalar-product method at $\sqrt{\smash [b]{s_{_{\mathrm {NN}}}}} = 2.76$ and 5.02$\,\text {TeV}$, as a function of centrality. Statistical (bars) and systematic (shaded boxes) uncertainties are shown. The results are compared with predictions at $\sqrt{\smash [b]{s_{_{\mathrm {NN}}}}} = 2.76\,\text {TeV}$ from AMPT [48] as well as hydrodynamics with a deformed symmetric Gaussian density profile as the initial conditions using $\eta /s = 0.08$ from Ref. [1], and from iEBE-VISHNU hydrodynamics with both Glauber and the KLN initial conditions using the same $\eta /s$ [28]

**Fig. 6:**
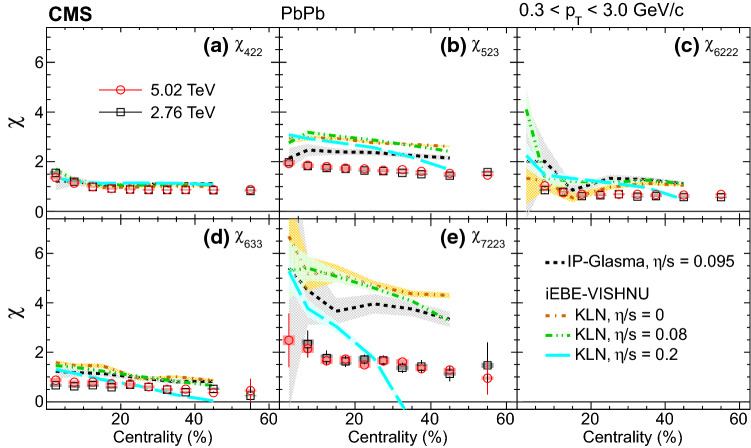
The same results as in Fig. 5 but compared with predictions from a hydrodynamics $+$ hadronic cascade hybrid approach with the IP-Glasma initial conditions using $\eta /s = 0.095$ [50] at $\sqrt{\smash [b]{s_{_{\mathrm {NN}}}}} = 5.02\,\text {TeV}$ and from iEBE-VISHNU hydrodynamics with the KLN initial conditions using $\eta /s = 0$, 0.08 (the same curve as in Fig. 5) and 0.2 [28] at $\sqrt{\smash [b]{s_{_{\mathrm {NN}}}}} = 2.76\,\text {TeV}$

The nonlinear response coefficients, $\chi _{422}$, $\chi _{523}$, $\chi _{6222}$, $\chi _{633}$, and $\chi _{7223}$ are presented in Figs. 5 and 6, as a function of centrality in the $p_{\mathrm {T}}$ range from 0.3 to 3.0${\,\text {GeV/}c}$. The results are compared with predictions at $\sqrt{\smash [b]{s_{_{\mathrm {NN}}}}} = 2.76\,\text {TeV}$ from the microscopic transport model AMPT [48, 49], a macroscopic hydrodynamic model using a deformed symmetric Gaussian density profile as the initial conditions with $\eta /s = 0.08$ [1], and from another hydrodynamic calculation (iEBE-VISHNU) with both Glauber and Kharzeev–Levin–Nardi (KLN) gluon saturation initial conditions using the same $\eta /s$ [28]. The model with Gaussian profile initial conditions gives a better description of the nonlinear response coefficients compared to other calculations, but it underestimates the values of $v_7\{\varPsi _{223}\}$ for centrality above 30%, as shown in Fig. 4. In Fig. 6, the same results are compared with the predictions from hydrodynamics $+$ hadronic cascade hybrid approach with the IP-Glasma initial conditions using $\eta /s = 0.095$ [50] at $\sqrt{\smash [b]{s_{_{\mathrm {NN}}}}} = 5.02\,\text {TeV}$ and from iEBE-VISHNU hydrodynamics with the KLN initial conditions using $\eta /s = 0$, 0.08 and 0.2 [28] at $\sqrt{\smash [b]{s_{_{\mathrm {NN}}}}} = 2.76\,\text {TeV}$. All the calculations describe the $\chi _{422}$ well, but none of them are successful for $\chi _{523}$ and $\chi _{7223}$. The model calculations of $\chi _{7223}$ are quite different for various initial conditions and $\eta /s$, which suggests that the first-time measurement of $\chi _{7223}$ presented in this paper could provide strong constraints on models.

## Summary

The mixed higher-order flow harmonics and nonlinear response coefficients of charged particles have been studied as functions of transverse momentum $p_{\mathrm {T}}$ and centrality in $\mathrm {PbPb}$ collisions at $\sqrt{\smash [b]{s_{_{\mathrm {NN}}}}} = 2.76$ and 5.02$\,\text {TeV}$ using the CMS detector. The measurements use the scalar-product method, covering a $p_{\mathrm {T}}$ range from 0.3 to 8.0${\,\text {GeV/}c}$, pseudorapidity $|\eta |<0.8$, and a centrality range of 0–60%. The mixed higher-order flow harmonics, $v_4\{\varPsi _{22}\}$, $v_5\{\varPsi _{23}\}$, $v_6\{\varPsi _{222}\}$, $v_6\{\varPsi _{33}\}$, and $v_7\{\varPsi _{223}\}$ all have a qualitatively similar $p_{\mathrm {T}}$ dependence, first increasing at low $p_{\mathrm {T}}$, reaching a maximum at about 3–4${\,\text {GeV/}c}$, and then decreasing at higher $p_{\mathrm {T}}$. As a comparison, the overall $v_n$ harmonics ($n = 4$–7) with respect to their own symmetry planes are measured in the same $p_{\mathrm {T}}$, $\eta$, and centrality ranges. The relative contribution of the nonlinear part for $v_5$ is larger than for other harmonics in the centrality range 20–60%. In addition, the nonlinear response coefficients of the odd harmonics are observed to be larger than those of even harmonics for $p_{\mathrm {T}}$ less than 3${\,\text {GeV/}c}$. At $p_{\mathrm {T}}$ less than 1${\,\text {GeV/}c}$, a viscous hydrodynamic calculation with Glauber initial conditions and shear viscosity to entropy density ratio $\eta /s = 0.08$ predicts a much stronger $p_{\mathrm {T}}$ dependence for the nonlinear response coefficients. The coefficients, including the first-time measurement of $\chi _{7223}$, as a function of centrality, are compared with AMPT and hydrodynamic predictions using different $\eta /s$ and initial conditions. Compared to the data, none of the models provides a simultaneous description of the mixed higher-order flow harmonics and nonlinear response coefficients. Therefore, these results can constrain both initial conditions and transport properties of the produced medium.

## Data Availability

This manuscript has no associated data or the data will not be deposited. [Authors’ comment: Release and preservation of data used by the CMS Collaboration as the basis for publications is guided by the CMS policy as written in its document “CMS data preservation, re-use and open access policy” (https://cms-docdb.cern.ch/cgi-bin/PublicDocDB/RetrieveFile?docid=6032&filename=CMSDataPolicyV1.2.pdf&version=2).]
